# Pediatric Tuberculosis Outside a Specialized TB Center: Clinical Presentation, Diagnostic Pathways, and Factors Associated with Microbiological Confirmation in a Romanian Tertiary Hospital

**DOI:** 10.3390/microorganisms14092042

**Published:** 2026-09-13

**Authors:** Alexandru-Ioan Ulmeanu, Alexandru Dinulescu, Andrei-Vlad Totu, Ion Alexandru Voropanov

**Affiliations:** 1 Department of Pediatrics, “Carol Davila” University of Medicine and Pharmacy, 020021 Bucharest, Romania; alexandru.ulmeanu@umfcd.ro (A.-I.U.); andrei-vlad.totu0125@rez.umfcd.ro (A.-V.T.); ion-alexandru.voropanov@drd.umfcd.ro (I.A.V.); 2 Department of Pediatrics, Emergency Hospital for Children ‘’Grigore Alexandrescu”, 011743 Bucharest, Romania; 3 Department of Pediatric Pneumology, “Marius Nasta” Institute of Pneumoftiziology, 050159 Bucharest, Romania

**Keywords:** tuberculosis, pediatric tuberculosis, bacteriologically confirmed TB, tuberculin skin test, QuantiFERON-TB Gold Plus, GeneXpert MTB/RIF

## Abstract

Pediatric tuberculosis (TB) remains diagnostically challenging because of nonspecific presentations and the frequent absence of microbiological confirmation. We aimed to characterize pediatric TB patients presenting to a non-specialized tertiary hospital in Romania and to explore factors associated with bacteriological confirmation. We retrospectively analyzed children diagnosed with TB between 2017 and 2025 at a tertiary pediatric hospital in Bucharest. Clinical, epidemiological, laboratory, radiological, microbiological, and follow-up data were evaluated. Factors associated with bacteriological confirmation were assessed using univariate and exploratory multivariable analyses. Among 48 patients, the median age was 14 years (IQR 8.25–16), and 37.5% of cases were bacteriologically confirmed. Initial presentations were pleural effusion (47.9%), pneumonia (41.7%), and cavitary disease (10.4%). No patient with pleural effusion had a documented TB contact, compared with 45.0% with pneumonia and 40.0% with cavitary disease (*p* < 0.001). Bacteriologically confirmed patients were older and more frequently had cavitary lesions. In exploratory Firth penalized logistic regression, older age and the presence of cavitary lesions remained associated with bacteriological confirmation. Pediatric TB may mimic common respiratory diseases even without known TB contact. In exploratory Firth penalized logistic regression, older age and cavitary lesions were associated with bacteriological confirmation, although the estimates, particularly for cavitary lesions, were imprecise and should be interpreted cautiously, while the substantial proportion of clinically diagnosed cases highlights the importance of integrating clinical, radiological, immunological, and microbiological findings.

## 1. Introduction

Tuberculosis (TB) remains one of the leading infectious causes of morbidity and mortality worldwide, and according to the World Health Organization (WHO) report from 2024, an estimated 10 million people develop tuberculosis each year, with over one million deaths globally [1,2,3]. Children account for a substantial proportion of the global burden, with it being estimated that more than one million children develop TB annually, representing approximately 10–12% of all cases worldwide, with the estimated real incidence in children being almost double the number of reported cases [3,4]. The burden is disproportionately higher in low- and middle-income countries, where delayed diagnosis, limited access to microbiological confirmation, and underreporting contribute to significant gaps in case detection [5,6,7,8].

The clinical presentation of tuberculosis in children differs substantially from that observed in adults, with pediatric TB being frequently paucibacillary, making microbiological confirmation more challenging [9,10,11,12]. Symptoms are often nonspecific and may include prolonged fever, chronic cough, weight loss, fatigue, or failure to thrive, also making clinical diagnosis difficult [13,14]. In younger children, especially infants and toddlers, respiratory symptoms may be mild or absent, while systemic manifestations pre-dominate, and infants are especially vulnerable to rapid disease progression following primary infection, with an increased risk of developing severe or disseminated forms such as miliary TB or tuberculous meningitis [6,15,16,17]. Intrathoracic lymphadenopathy is a common radiological finding, whereas cavitary disease is less frequent than in adult patients [18,19,20]. Extrapulmonary tuberculosis occurs more commonly in children and may involve lymph nodes, the central nervous system, bones, or serous membranes [6,21,22]. Atypical presentations such as persistent pneumonia unresponsive to broad-spectrum antibiotics, prolonged fever of unknown origin, isolated lymphadenopathy, or neurological symptoms often delay diagnosis of TB even more [23,24,25]. These diagnostic challenges contribute to underdiagnosis and may influence the likelihood of obtaining microbiological confirmation.

Despite a steady decline over the past few decades due to national control programs aligned with international strategies promoted by the World Health Organization and implemented through the national TB surveillance system, Romania remains a high-burden setting compared with other EU member states [26,27,28]. The last available surveillance report published by the European Centre for Disease Prevention and Control (ECDC) is from 2022, and it reported that Romania alone was responsible for 25.6% of all TB cases reported in the European Union (EU) in that year [29]. Adult TB accounts for the majority of notified cases, with pediatric cases representing a smaller but epidemiologically significant component of the national burden [30,31,32].

The objective of this study was to characterize the clinical and diagnostic pathways of pediatric tuberculosis in a tertiary non-specialized pediatric hospital in Romania and to explore factors associated with bacteriological confirmation.

## 2. Materials and Methods

### 2.1. Study Settings

This retrospective observational study was conducted at “Grigore Alexandrescu” Emergency Clinical Hospital for Children, a tertiary pediatric hospital in Romania that is not specialized in tuberculosis management. The hospital serves as a referral center for a wide range of pediatric pathologies, including infectious, respiratory, neurological, and systemic diseases.

Children with tuberculosis are frequently initially admitted with suspected alternative diagnoses (e.g., pneumonia, prolonged fever of unknown origin, lymphadenopathy, neurological conditions) before tuberculosis is considered. As the hospital features a tertiary pediatric pulmonology department, most children were initially admitted for the evaluation of other pulmonary conditions rather than suspected tuberculosis. The most frequent admission diagnoses included pleural effusion, pneumonia, and lung abscess. Tuberculosis was subsequently considered based on the clinical evolution of the disease, imaging findings, epidemiological history, and microbiological investigations, after which patients were referred to a specialized tuberculosis center. Once the diagnosis was established or strongly suspected, patients were referred to the “Marius Nasta” Institute of Pneumology, a specialized tuberculosis hospital, for continuation of treatment, according to national TB control regulations. After the patients were referred to the specialized tuberculosis hospital, we monitored them for confirming the diagnosis. Follow-up information from the referral center was reviewed to ascertain the final diagnosis and microbiological confirmation status.

### 2.2. Study Population

All patients aged 0–18 years diagnosed with tuberculosis between 1 January 2017 and 31 December 2025 were eligible for inclusion. This timeframe was selected because electronic medical records were consistently available starting in 2017, following the implementation of a new hospital information system, which allowed for reliable and comprehensive data retrieval.

Inclusion criteria include the following:

- Diagnosis of active pulmonary TB established during hospitalization or shortly thereafter;
- Availability of complete medical records.

Exclusion criteria included the following:

- Incomplete clinical or laboratory data;
- Cases in which TB diagnosis could not be confirmed or reasonably established based on national guidelines.

**Figure 1:**
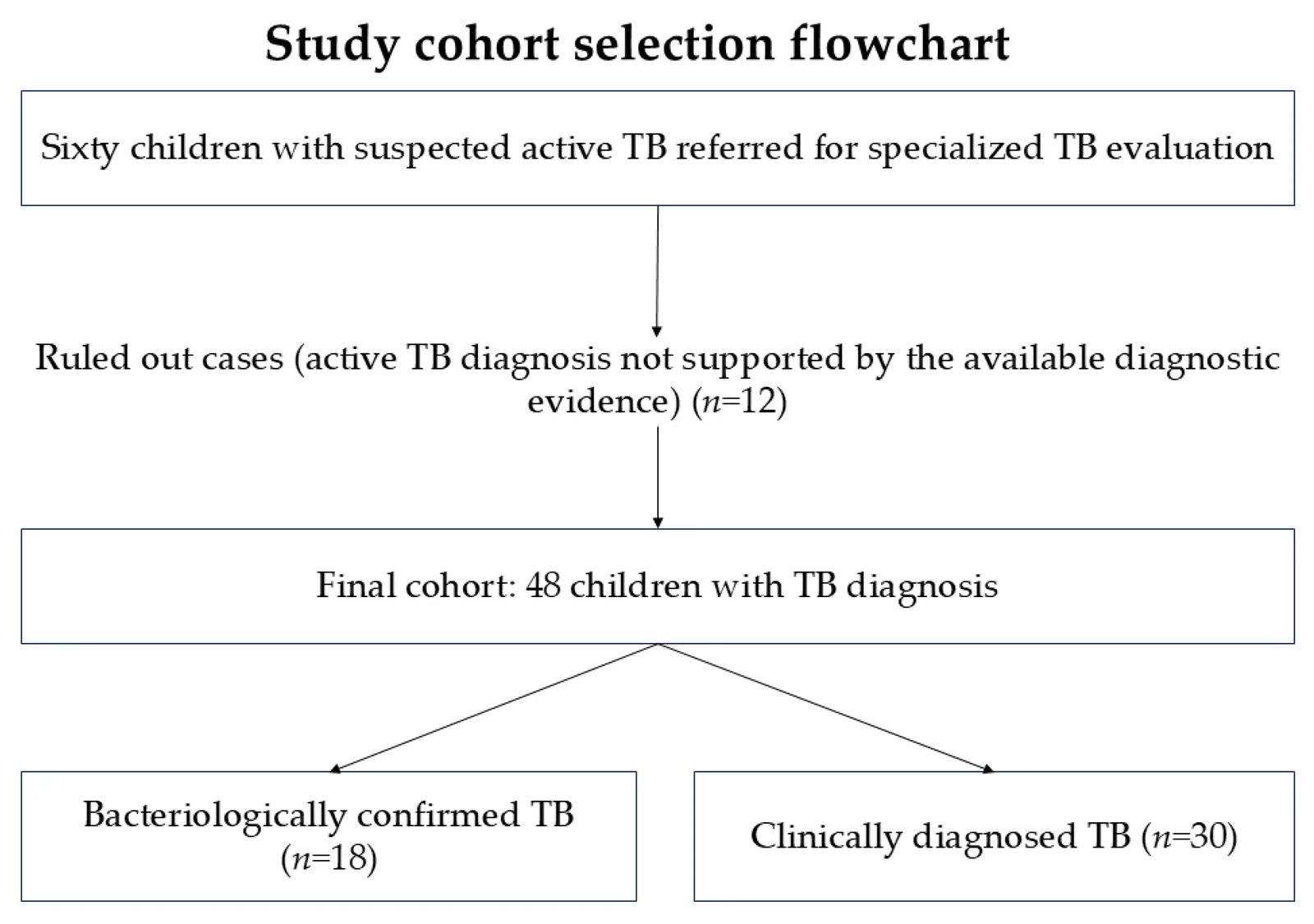
Study cohort selection.

A total of 60 children in whom active TB was suspected during hospitalization were referred to the specialized tuberculosis center for further evaluation. Following assessment by pediatric TB specialists at the “Marius Nasta” Institute of Pneumology, active TB was considered not to be supported by the available diagnostic evidence in 12 children, and these patients were therefore excluded from the present study. The retrospective records available to our center did not contain sufficiently detailed information to further categorize the individual reasons for exclusion. The remaining 48 children, in whom the diagnosis of active TB was retained by the specialized TB center, constituted the final study cohort (Figure 1).

### 2.3. Definitions and Diagnostic Classification

Tuberculosis was suspected during hospitalization based on the integration of clinical, epidemiological, and radiological findings suggestive of active TB. Patients in whom TB was suspected underwent further diagnostic evaluation, which could include the tuberculin skin test (TST), QuantiFERON-TB Gold Plus, and microbiological investigations (smear microscopy, mycobacterial culture, and/or GeneXpert MTB/RIF), according to clinical indication and feasibility. Patients were subsequently referred to the specialized tuberculosis center for further evaluation. The final diagnosis established by the pediatric TB specialists at the referral center was used for study inclusion and diagnostic classification. Of the 60 children initially referred with suspected active TB, active TB was not supported by the available diagnostic evidence after specialized evaluation in 12 patients, who were therefore excluded. The TB cases were classified according to the World Health Organization criteria [33] as follows:

- Bacteriologically confirmed TB: detection of Mycobacterium tuberculosis by smear microscopy, culture, or molecular methods.
- Clinically diagnosed TB: cases without microbiological confirmation but with compatible clinical, radiological, epidemiological, and/or immunological findings leading to the initiation of anti-TB treatment

The clinically diagnosed TB cases were confirmed by pediatric TB specialists at the “Marius Nasta” Institute of Pneumology based on an integrated assessment of compatible clinical and radiological findings, with epidemiological and/or immunological evidence considered supportive when available, and for whom anti-TB treatment was initiated.

For analysis of the initial presentation, patients were classified into three mutually exclusive clinical–radiological groups according to the predominant presentation leading to admission: pleural effusion, pneumonia, or cavitary pulmonary disease. When multiple radiological abnormalities coexisted, classification was based on the predominant clinical–radiological presentation documented at admission.

Fever was defined as a body temperature higher than 38.0 °C (100.4 °F), in accordance with the guidelines of the European Centre for Pediatric and Adolescent Medicine (ECPA) and the American Academy of Pediatrics (AAP) [34,35].

The tuberculin skin test (TST) was considered positive if the induration was 10 mm or more (5TU) [14,36].

The cutoff value for a positive QuantiFERON-TB Gold Plus was 0.35 IU/mL [37].

Laboratory specimens were collected and analyzed during hospitalization. This study is retrospective and the results were available in the electronic medical records. No biological samples were stored or reanalyzed for research purposes. Microbiological investigations included smear microscopy, mycobacterial culture, and GeneXpert MTB/RIF and were performed according to clinical indication and feasibility rather than according to a uniform study protocol. Smear microscopy and mycobacterial culture were performed in all 48 patients. GeneXpert MTB/RIF was performed in 22 patients. Molecular testing was not systematically available throughout the entire study period because of changes in the local availability of the assay and occasional shortages of testing kits at the specialized TB center. Therefore, GeneXpert results were available only for a subset of the cohort. The present analysis therefore evaluates factors associated with documented bacteriological confirmation within the local diagnostic pathway rather than the diagnostic performance of individual microbiological methods.

Chest radiography was the primary imaging modality and was performed in all patients. Additional imaging, including chest computed tomography and/or lung ultrasonography, was performed selectively according to clinical indications; however, these investigations were not performed systematically across the cohort and were therefore not included in the comparative analyses. Radiographic findings were initially obtained from the original clinical radiology reports, which were interpreted by radiologists as part of routine clinical care. All radiographic images were subsequently reviewed by the study investigators to verify the reported findings and ensure consistent classification for the purposes of the study.

For analysis of the initial presentation, patients were classified into three mutually exclusive clinical–radiological groups according to the predominant presentation leading to admission: pleural effusion, pneumonia, or cavitary pulmonary disease. When multiple abnormalities coexisted, patients were assigned to the group corresponding to the predominant clinical–radiological presentation documented at admission. These presentation groups were used to characterize the principal syndrome at admission and were distinct from the individual radiological abnormalities analyzed separately.

Known TB contact was based on the medical history recorded at admission and was defined as a patient- or caregiver-reported history of contact with a person known to have tuberculosis. In the absence of such a reported exposure, the patient was classified as having no known TB contact. BCG vaccination status was obtained from the child’s vaccination record.

All patients included in this study underwent HIV testing, and all had negative results.

### 2.4. Statistical Analysis

Statistical analysis was performed using IBM SPSS Statistics version 25 (IBM Corp., Armonk, NY, USA). Quantitative variables were tested for normal distribution using the Shapiro–Wilk test, normally distributed variables were expressed as mean ± standard deviation (SD), and non-normally distributed variables were reported as median and interquartile range (IQR). Quantitative variables were tested between independent groups using Mann–Whitney U tests. Fisher’s exact test was used to determine the nonrandom associations between categorical variables, with the Bonferroni method used for correction. Factors associated with bacteriological confirmation were initially explored using univariate analyses. Given the limited number of bacteriologically confirmed cases and the sparse distribution of cavitary lesions, an exploratory multivariable analysis was subsequently performed using Firth penalized logistic regression to reduce small-sample bias and address potential quasi-separation. Bacteriological confirmation was used as the dependent variable, and the model was deliberately restricted to age and the presence of cavitary lesions to minimize overfitting. These variables were selected based on their clinical relevance and their associations in univariate analysis. Penalized odds ratios (ORs) with 95% confidence intervals (CIs) were reported. The multivariable analysis was considered exploratory rather than predictive. Given the number of univariate comparisons and the limited sample size, the univariate analyses were also considered exploratory, and findings with marginal *p*-values were interpreted cautiously. A *p*-value < 0.05 was considered statistically significant.

## 3. Results

### 3.1. Subject Characteristics

A total of 48 patients were included in this study. The distribution of cases by year of diagnosis showed variability over time, with the highest number of cases being recorded in 2020 (16.7%), followed by 2021 (14.6%) and 2019 (12.5%). In the remaining years, the proportion of cases ranged between 6.3% and 10.4%. Regarding seasonal variation, most cases were diagnosed during spring (33.3%) and summer (29.2%), while fewer cases occurred in fall and winter (both 18.8%). The study population was evenly distributed by sex, with 24 males (50%) and 24 females (50%). The median age at admission was 14 years (8.25–16). The majority of patients originated from rural areas (68.8%), whereas 31.2% were from urban areas. Most of them (95.8%) were vaccinated for TB. Less than a quarter of them (22.9%) described a known TB contact (Table 1.). No documented HIV infection cases, chronic pulmonary diseases, or immunodeficiencies were identified in the available records, and no MDR/XDR tuberculosis cases were identified. All 48 included patients had pulmonary and/or intrathoracic TB; no extrapulmonary involvement was identified in the study cohort.

### 3.2. Clinical Presentation

The most common clinical manifestation at presentation was cough (85.4%), followed by fever (77.1%). Among those with fever, the mean temperature was 39.01 ± 0.57 °C, and the median duration of fever was 5 days (IQR: 4–7). Respiratory symptoms were variably present: chest pain occurred in 41.7% of cases, dyspnea in 22.9% of cases, and hemoptysis in only 4.2% of patients. General symptoms included fatigue in 29.2% of patients, weight loss in 16.7% of patients, and nocturnal diaphoresis in 10.4% of patients. On physical examination, diminished vesicular breath sounds were the most frequent finding (66.7%), followed by crackles (39.6%) and intercostal retractions (20.8%). Lymphadenopathies were identified in 10.4% of patients, while hepatosplenomegaly was uncommon (2.1%). The median duration of hospitalization until transfer to the referral center was 6 days (IQR: 4–8) (Table 2.).

### 3.3. Laboratory Findings

The median white blood cell count was 9.6 × 10^9^/L (7.95–12.82), with a median neutrophil count of 6.67 × 10^9^/L (4.33–8.24) and a median lymphocyte count of 1.94 × 10^9^/L (1.55–3.18). The median monocyte count was 0.959 × 10^9^/L (0.58–1.28). The median hemoglobin level was 11.9 g/dL (10.22–12.67). C-reactive protein (CRP) had a median of 6.2 (2.54–12.32) mg/dL. TST was positive in 62.5% of patients. QuantiFERON-TB Gold Plus testing was performed in 34 patients (70.8%), with a positivity rate of 88.2% among those tested. The median QuantiFERON-TB Gold Plus value was 1.61 IU/mL (0.66–3.27) overall and 1.74 IU/mL (0.72–3.64) in patients with positive results. Molecular testing using the GeneXpert assay was performed in 45.8% of patients, of whom 54.5% had positive results. Positive smear microscopy was identified in 25% of cases, while Mycobacterium tuberculosis cultures were positive in 22.9% of patients (Table 3.).

### 3.4. Chest X-Ray Findings

All of the patients had a chest X-ray performed on them at admission. The most frequent radiological abnormalities were pulmonary infiltrates, identified in 62.5% of patients, followed by consolidation in 58.3% of cases and pleural effusion in 47.9% of cases. Cavitary lesions and mediastinal or hilar lymphadenopathies were less common, with each being present in 16.7% of patients (Table 4).

The presence of cough, fever, weight loss, nocturnal diaphoresis, fatigue, hemoptysis, and dyspnea was not associated with any radiological aspect (*p* > 0.05).

Pleural effusion on chest radiography was significantly more common among patients presenting with chest pain compared to those without chest pain (70.0% vs. 32.1%, *p* = 0.018), as well as in those with diminished vesicular breath sounds (65.6% vs. 12.5%, *p* = 0.001), and it was significantly less common among patients presenting with crackles compared to those without crackles (26.3% vs. 62.1%, *p* = 0.020).

Pulmonary consolidation on chest radiography was significantly more common among patients presenting with crackles compared to those without crackles (78.9% vs. 44.8%, *p* = 0.035).

Mediastinal or hilar lymphadenopathies on chest radiography were significantly more common among patients presenting with peripheral lymphadenopathies (80.0% vs. 9.3%, *p* = 0.002).

### 3.5. Clinical Phenotypes According to Initial Presentation

Patients were classified into three mutually exclusive groups according to their predominant clinical–radiological presentation at admission: pleural effusion (*n* = 23, 47.9%), pneumonia (*n* = 20, 41.7%), and cavitary pulmonary disease (*n* = 5, 10.4%). Age and sex distribution did not differ significantly among the three groups (*p* > 0.05). The results for a known tuberculosis contact differed significantly according to the initial presentation (*p* < 0.001). None of the patients presenting with pleural effusion had a documented TB contact, compared with 45.0% of those presenting with pneumonia and 40.0% of those with cavitary disease. In pairwise analysis, the difference between the pleural effusion and pneumonia groups remained significant after Bonferroni correction (adjusted *p* < 0.001).

Clinical findings also differed across presentation groups. Cough was more frequent in patients with pleural effusion (95.7%) and pneumonia (85.0%) than in those with cavitary disease (40.0%) (*p* = 0.013), with the difference between the pleural and cavitary groups remaining significant after Bonferroni correction (adjusted *p* = 0.034). Diminished vesicular breath sounds were particularly frequent in the pleural effusion group, with an occurrence rate of 91.3%, compared with 45.0% in the pneumonia group and 40.0% in the cavitary group (*p* < 0.001); the difference between pleural effusion and pneumonia remained significant after correction (adjusted *p* = 0.006). Conversely, crackles were more frequent in patients presenting with pneumonia than in those with pleural effusion (60.0% vs. 21.7%; adjusted *p* = 0.043). Peripheral lymphadenopathy was observed exclusively among patients presenting with pneumonia (25.0%; global *p* = 0.023). CRP levels also differed significantly among the three groups (*p* = 0.015), with higher values recorded in the pleural effusion group than in the pneumonia group [10.42 (6.19–13.07) vs. 3.83 (2.31–6.27) mg/dL; adjusted *p* = 0.007].

Microbiological findings varied according to the initial presentation. Bacteriological confirmation was obtained in 80.0% of patients with cavitary disease, compared with 35.0% of those presenting with pneumonia and 30.4% of those with pleural effusion; however, this difference did not reach statistical significance (*p* = 0.153). Culture positivity differed significantly among the three groups (*p* = 0.004), being highest in patients with cavitary disease, with an occurrence rate of 80.0%, compared with 25.0% in the pneumonia group and 8.7% in the pleural effusion group. The difference between the cavitary and pleural effusion groups remained significant after Bonferroni correction (adjusted *p* = 0.010) (Table 5).

### 3.6. Factors Associated with Bacteriological Confirmation

Of the 48 patients included in this study, 18 (37.5%) had bacteriologically confirmed tuberculosis, while 30 (62.5%) were clinically diagnosed. Patients with bacteriologically confirmed tuberculosis were significantly older than those with clinically diagnosed disease [median 15.5 (IQR 12.75–16.25) vs. 12.5 (IQR 3–15) years, *p* = 0.006]. No significant differences were observed according to sex, area of residence, BCG vaccination status, known TB contact, or season of diagnosis (all *p* > 0.05).

Weight loss was significantly more frequent among bacteriologically confirmed patients than among clinically diagnosed patients (33.3% vs. 6.7%, *p* = 0.040). No significant differences were observed for the remaining clinical manifestations (all *p* > 0.05). Bacteriologically confirmed patients had significantly lower peripheral lymphocyte counts [median 1.62 (IQR 1.43–2.01) vs. 2.28 (IQR 1.76–3.50) × 10^9^/L, *p* = 0.020], whereas the remaining routinely assessed laboratory parameters did not differ significantly between groups. Neither TST positivity nor QuantiFERON-TB Gold Plus results were significantly associated with bacteriological confirmation (*p* > 0.05).

Cavitary lesions were markedly more frequent among bacteriologically confirmed patients than among clinically diagnosed patients (38.9% vs. 3.3%, *p* = 0.003). No significant associations were identified between bacteriological confirmation and the other chest radiographic findings (all *p* > 0.05) (Table 6).

Given the small number of bacteriologically confirmed cases and the sparse distribution of cavitary lesions, Firth penalized logistic regression was used for the exploratory multivariable analysis. Increasing age remained associated with bacteriological confirmation (OR 1.18 per additional year, 95% CI 1.03–1.43; *p* = 0.014). The presence of cavitary lesions was also associated with higher odds of bacteriological confirmation (OR 15.79, 95% CI 2.48–233.83; *p* = 0.002) (Table 7.). The wide confidence interval for cavitary lesions reflects the small number of patients with this finding and warrants cautious interpretation.

## 4. Discussion

This is a retrospective study that describes the real-world presentation and diagnostic pathway of pediatric TB in a tertiary pediatric hospital that is not specialized in tuberculosis care. None of the included children had a diagnosis of tuberculosis at admission. This setting is clinically relevant because pediatric tuberculosis frequently presents with nonspecific manifestations and microbiological confirmation remains difficult, particularly in paucibacillary disease [8,9,10,11,12,13,14]. In our cohort, only 37.5% of cases were bacteriologically confirmed, despite the predominance of older children and adolescents, showing that even in age groups more likely to produce respiratory specimens and develop adult-type pulmonary disease, diagnosis still often relies on the integration of epidemiological, clinical, radiological, immunological, and microbiological findings [38,39,40]. The 37.5% proportion is comparable to the 44% microbiological confirmation rate reported in a retrospective cohort of 151 children with TB published by Indumathi et al. (2022), while other pediatric studies report confirmation rates ranging from approximately 25% to over 50% [41,42,43].

We found a heterogeneous initial clinical–radiological presentation. Pleural effusion was the most frequent presentation, being present in 47.9% of cases, followed by pneumonia in 41.7% of cases and cavitary pulmonary disease in 10.4% of cases. Pleural TB is recognized more frequently in older children and adolescents and may reflect a more mature delayed hypersensitivity response to Mycobacterium tuberculosis [6,16,44]. It is worth mentioning that none of the children presenting with pleural effusion had a documented history of known TB contact, compared with 45.0% of those presenting with pneumonia and 40.0% of those with cavitary disease, even though childhood tuberculosis is often acquired from household or other close exposure to an infectious adult, with epidemiological history being a central component of TB diagnosis [16,45]. A recognized source of exposure is also absent in a substantial proportion of pediatric pleural TB cases reported elsewhere: contact with an infectious TB case was identified in 44% of patients in a US series of 45 children with pleural TB and in only 13% of patients in a Chinese cohort of 154 children [46,47]. The absence of a recognized contact cannot reliably exclude tuberculosis, and this may be especially relevant for pediatricians working outside dedicated tuberculosis services, where the initial presentation may resemble more common respiratory disorders.

The initial presentation groups also showed distinct clinical profiles. Children with pleural effusion more frequently had diminished vesicular breath sounds and higher CRP concentrations, whereas crackles were more common in those presenting with pneumonia. These findings are clinically coherent with the underlying radiological patterns and support the value of integrating physical examination with imaging findings. The cavitary group was small, but it showed the highest proportion of bacteriological confirmation and a higher rate of positive mycobacterial culture. Culture was positive in 80.0% of children with cavitary presentation, compared with 25.0% of those with pneumonia and 8.7% of those with pleural effusion. Comparable findings were reported by Uşaklıoğlu Erol et al. (2026) in a cohort of two hundred and fifty children with pulmonary TB, among whom eight had cavitary disease; six of these eight patients (75%) had positive cultures and four (50%) had positive smear microscopy [48]. Cavitary lesions are known to contain substantially higher mycobacterial burdens and to facilitate bacillary discarding into respiratory secretions, thereby increasing the diagnostic rate of smear microscopy, culture, and molecular assays [49,50,51]. Although the small number of children with cavitary disease limits precise estimation, this pattern was consistent with the biological relationship between cavitation and higher bacillary burden.

Bacteriological confirmation was associated with older age, weight loss, lower lymphocyte counts, and cavitary lesions. Older children and adolescents are more likely to develop post-primary or adult-type pulmonary tuberculosis, including cavitary disease, and they are also more frequently able to provide spontaneous sputum specimens, both of which may improve microbiological diagnosis [16,44,52]. In the exploratory Firth penalized multivariable analysis, increasing age remained independently associated with bacteriological confirmation, with a 18% increase in the odds of confirmation for each additional year of age. Cavitary lesions were also independently associated with substantially higher odds of bacteriological confirmation. The very wide confidence interval around this estimate should, however, be interpreted cautiously because only eight patients had cavitary lesions. These findings should therefore be considered exploratory and hypothesis-generating rather than components of a predictive diagnostic model.

Weight loss was more frequent among bacteriologically confirmed patients, which is consistent with previous observations that constitutional symptoms may be more prominent in patients with greater disease burden [40,53]. Similarly, bacteriologically confirmed children had lower lymphocyte counts than clinically diagnosed patients. Lymphopenia has been described in active and more severe tuberculosis and may reflect lymphocyte redistribution, impaired T-cell proliferation, and increased apoptosis during active infection [54,55,56]. Neither TST nor QuantiFERON-TB Gold Plus differentiated between clinically diagnosed and bacteriologically confirmed disease. This is biologically expected, as both tests primarily reflect immunological sensitization to Mycobacterium tuberculosis rather than bacillary burden or disease severity [49,57,58]. QuantiFERON-TB Gold Plus was positive in 88.2% of tested patients and therefore remained a useful supportive component of the diagnostic work-up, particularly when microbiological confirmation could not be obtained [59,60,61,62].

From a practical perspective, our findings emphasize that tuberculosis should remain under consideration in the differential diagnosis of children and adolescents presenting to tertiary pediatric services with unexplained pleural effusion, persistent pneumonia, cavitary pulmonary disease, prolonged systemic symptoms, or suggestive radiological abnormalities, even when a known TB contact is absent [9,52,63]. The large proportion of clinically diagnosed cases illustrates the continued importance of integrated diagnostic assessment in pediatric tuberculosis and the limitations of relying exclusively on microbiological confirmation.

The highest annual numbers of TB cases in our cohort were observed in 2020 (*n* = 8) and 2021 (*n* = 7), coinciding with the COVID-19 pandemic. Although the pandemic substantially disrupted TB diagnostic and control services worldwide, our study was not designed to evaluate temporal trends or the impact of the pandemic on pediatric TB [64,65,66]. Given the small annual case numbers and the single-center design, this observation should be interpreted descriptively and cannot be attributed to pandemic-related changes.

### Limitations

This study has several strengths. It describes pediatric TB from the perspective of a non-specialized tertiary pediatric hospital, capturing patients before referral to specialized TB care and therefore reflecting the diagnostic challenges encountered in general pediatric practice. None of the included children had an established diagnosis of TB at initial admission, and the nine-year study period allowed the characterization of a relatively uncommon disease across a broad range of clinical presentations.

Several limitations of this study should be acknowledged. The retrospective design introduced the possibility of incomplete or inconsistent data collection, particularly regarding nutritional parameters and use of molecular testing. This was a single-center study performed in a non-specialized tertiary hospital, which limits the generalizability of the findings to the broader pediatric TB population in Romania; therefore, our findings should be interpreted as representative of children referred to a tertiary pediatric center rather than the entire pediatric population of Romania. The relatively small sample size limited the number of variables that could be included in the multivariable analysis and reduced the precision of the estimates, particularly for infrequent findings such as cavitary lesions. Another important limitation is that microbiological investigations were performed as part of routine clinical care rather than according to a standardized prospective study protocol. Smear microscopy and mycobacterial culture were performed in all patients, whereas GeneXpert MTB/RIF was available only for a subset of the cohort, partly because of limited assay availability during the earlier years of the study and occasional kit shortages. In addition, detailed information regarding specimen type and sampling procedures could not be consistently reconstructed from the retrospective records. These factors may have influenced the likelihood of documented bacteriological confirmation and should be considered when interpreting the observed associations.

## 5. Conclusions

Pediatric tuberculosis presenting outside specialized TB services frequently mimicked common respiratory conditions, particularly pleural effusion and pneumonia. A documented TB contact was absent in most patients and in all children presenting predominantly with pleural effusion, emphasizing that the absence of recognized exposure should not rule out consideration of TB. In exploratory Firth penalized logistic regression, older age and cavitary lesions were associated with bacteriological confirmation, although the estimates, particularly for cavitary lesions, were imprecise and should be interpreted cautiously, while the substantial proportion of clinically diagnosed cases highlights the importance of an integrated clinical, radiological, immunological, and microbiological diagnostic approach.

## Figures and Tables

**Table 1:** Descriptive statistics of the population’s main characteristics.

Variable	*n* (%)	Median (IQR)
**Year of diagnosis**		
2017	5 (10.4%)	
2018	5 (10.4%)	
2019	6 (12.5%)	
2020	8 (16.7%)	
2021	7 (14.6%)	
2022	4 (8.3%)	
2023	3 (6.3%)	
2024	5 (10.4%)	
2025	5 (10.4%)	
**Season**		
Spring	16 (33.3%)	
Summer	14 (29.2%)	
Fall	9 (18.8%)	
Winter	9 (18.8%)	
**Sex**		
Male	24 (50%)	
Female	24 (50%)	
**Age (years)**		
		14 (8.25–16)
**Area of provenance**		
Urban	15 (31.2%)	
Rural	33 (68.8%)	
	**TB vaccination status**	
Vaccinated	46 (95.8%)	
Non-vaccinated	2 (4.2%)	
	**Known TB contact**	
Yes	11 (22.9%)	
No	37 (77.1%)	
	**Type of diagnosis**	
Clinically diagnosed TB	30 (62.5%)	
Bacteriologically confirmed TB	18 (37.5%)	

**Table 2:** Clinical characteristics.

Variable	*n* (%)	Median (IQR)/Average (SD)
Cough	41 (85.4%)	
Chest pain	20 (41.7%)	
Weight loss	8 (16.7%)	
Fever	37 (77.1%)	
Temperature of those with fever		39.01 ± 0.57°
Days of fever		5 (4–7)
Fatigue	14 (29.2%)	
Hemoptysis	2 (4,2%)	
Nocturnal diaphoresis	5 (10.4%)	
Dyspnea	11 (22.9%)	
Intercostal retractions	10 (20.8%)	
Diminished vesicular breath sounds	32 (66.7%)	
Crackles	19 (39.6%)	
Lymphadenopathies	5 (10.4%)	
Hepatosplenomegaly	1 (2.1%)	
Days of hospitalization until transfer to the referral center		6 (4–8)

**Table 3:** Lab findings.

Variable	*n* (%)	Median (IQR)/Average (SD)
WBC (×10^9^/L)		9.6 (7.95–12.82)
Neutrophils (×10^9^/L)		6.67 (4.33–8.24)
Lymphocytes (×10^9^/L)		1.94 (1.55–3.18)
Monocytes (×10^9^/L)		0.95 (0.58–1.28)
Hb (g/dL)		11.9 (10.22–12.67)
C-reactive protein (mg/dL)		6.2 (2.54–12.32)
Positive TST	30 (62.5%)	
QuantiFERON-TB Gold Plus (IU/mL) performed	34 (70.8%)	1.61 (0.66–3.27)
Positive QuantiFERON-TB Gold Plus (IU/mL)	30 (88.2%)	1.74 (0.72–3.64)
GeneXpert test performed	22 (45.8%)	
Positive GeneXpert test	12 (54.5%)	
Positive smear microscopy	12 (25%)	
Positive culture	11 (22.9%)	

**Table 4:** X-ray aspects.

Variable	*n* (%)
Consolidation	28 (58.3%)
Infiltrates	30 (62.5%)
Cavities	8 (16.7%)
Pleural effusion	23 (47.9%)
Lymphadenopathies	8 (16.7%)

**Table 5:** Clinical, epidemiological, and diagnostic characteristics categorized according to the initial clinical–radiological presentation of tuberculosis.

Characteristic	Pleural Effusion *n* = 23	Pneumonia *n* = 20	Cavitary Presentation *n* = 5	*p*
Age, years, median (IQR)	14 (11–16)	13 (3–15.25)	14 (9–15)	0.597
Known TB contact	0 (0%)	9 (45.0%)	2 (40.0%)	<0.001
Cough	22 (95.7%)	17 (85.0%)	2 (40.0%)	0.013
Chest pain	14 (60.9%)	6 (30.0%)	0	0.019
Fever	18 (78.3%)	16 (80.0%)	3 (60.0%)	0.693
Weight loss	4 (17.4%)	3 (15.0%)	1 (20.0%)	1.000
Diminished breath sounds	21 (91.3%)	9 (45.0%)	2 (40.0%)	<0.001
Crackles	5 (21.7%)	12 (60.0%)	2 (40.0%)	0.031
Peripheral lymphadenopathy	0	5 (25.0%)	0	0.023
CRP, mg/dL, median (IQR)	10.42 (6.19–13.07)	3.83 (2.31–6.27)	1.46 (1.37–18.15)	0.015
Bacteriologically confirmed TB	7 (30.4%)	7 (35.0%)	4 (80.0%)	0.153
Positive culture	2 (8.7%)	5 (25.0%)	4 (80.0%)	0.004

**Table 6:** Clinical, laboratory, and radiological characteristics categorized according to bacteriological confirmation status.

Variable	Clinically Diagnosed TB (*n* = 30)	Bacteriologically Confirmed TB (*n* = 18)	*p*-Value
**Epidemiological characteristics**			
Age, years, median (IQR)	12.5 (3–15)	15.5 (12.75–16.25)	**0.006**
Male sex, n (%)	12 (40.0%)	12 (66.7%)	0.135
BCG vaccinated, n (%)	28 (93.3%)	18 (100%)	0.521
Known TB contact, n (%)	6 (20.0%)	5 (27.8%)	0.724
**Clinical characteristics**			
Weight loss, n (%)	2 (6.7%)	6 (33.3%)	**0.040**
**Laboratory/immunological characteristics**			
Lymphocytes, ×10^9^/L, median (IQR)	2.28 (1.76–3.50)	1.62 (1.43–2.01)	**0.020**
TST positive, n (%)	16 (53.3%)	14 (77.8%)	0.127
QuantiFERON-TB Gold Plus positive, n/N (%)	18/21 (85.7%)	12/13 (92.3%)	1.000
**Radiological characteristics**			
Cavitary lesions, n (%)	1 (3.3%)	7 (38.9%)	**0.003**
**Residence**			0.757
Urban	10 (33.3%)	5 (27.8%)	
Rural	20 (66.7%)	13 (72.2%)	
**Season**			0.406
Spring	8 (26.7%)	8 (44.4%)	
Summer	11 (36.7%)	3 (16.7%)	
Fall	5 (16.7%)	4 (22.2%)	
Winter	6 (20.0%)	3 (16.7%)	

**Table 7:** Exploratory Firth penalized logistic regression analysis of factors associated with bacteriological confirmation.

Variable	OR	95% CI	*p*-Value
Age, per 1-year increase	1.18	1.03–1.43	0.014
Cavitary lesions	15.79	2.48–233.83	0.002

## Data Availability

Data are contained within the article.

## Abbreviations

The following abbreviations are used in this manuscript:

TB	Tuberculosis
IQR	Interquartile range
SD	Standard deviation
WHO	World Health Organization
